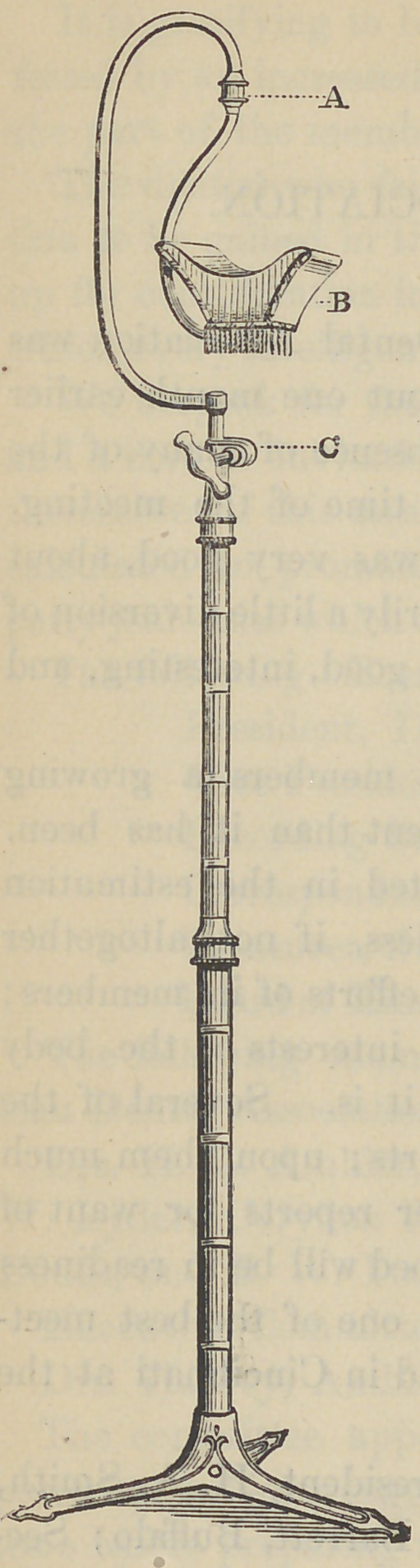# A New Arm Rest

**Published:** 1881-09

**Authors:** 


### A NEW ARM REST.

A recognized want by the dental profession, for a support for the left arm during the prolonged operation of filling teeth, induced one or two manufacturers of dental chairs to attach an extension to the left side of the head-rest, to serve as such a support. Its range of movement, however, is quite limited, and while it serves a good purpose in some cases, in others it is not only useless, but is in the way. To avoid this difficulty, and better meet the requirement, the stand with the adjustable rest attached, as shown in the accompanying illustration has been devised. It can be placed at any height, or put in any position required. It is the invention of Dr. E. Pierrepont, of London, England. For many operations it will be an invaluable appliance. They may be obtained through the dental depots.

## Figures and Tables

**Figure f1:**